# Effect of Jeju Water on Blood Glucose Levels in Diabetic Patients: A Randomized Controlled Trial

**DOI:** 10.1155/2013/212918

**Published:** 2013-11-18

**Authors:** Gwanpyo Koh, Dae Ho Lee, Sang Ah Lee, Eun-Kyung Kang, Okkyeong Hwang, Hyun-Jung Han, Sok Young Kim, Eun-Jin Yang, Min-Kyoung Kim, Hyoun-Jung Chin

**Affiliations:** ^1^Department of Internal Medicine, Jeju National University School of Medicine, 102 Jejudaehakno, Jeju City, Jeju 690-756, Republic of Korea; ^2^Department of Internal Medicine, College of Medicine, Wonkwang University, 460 Iksan-daero, Iksan-si, Jeollabuk-do 570-740, Republic of Korea; ^3^Jeju R&D center, BMI Korea, 2230-4 Yeongpyeong-dong, Jeju-si, Jeju-do 690-140, Republic of Korea; ^4^Department of Internal Medicine, Hankook General Hospital, 193 Seogwang-ro, Jeju City, Jeju 690-715, Republic of Korea

## Abstract

Jeju water is the groundwater of Jeju Island, a volcanic island located in Republic of Korea. We investigated whether Jeju water improved glycemic control in patients with diabetes. This was a 12-week single-center, double-blind, randomized, and controlled trial. The subjects daily drank a liter of one of three kinds of water: two Jeju waters (S1 and S2) and Seoul tap water (SS). The primary outcome was the proportion of patients in the per-protocol (PP) population achieving glycated hemoglobin (HbA1c) < 7.0% at week 12. In total, 196 patients were randomized and analyzed in the intention-to-treat (ITT) population (66 consuming S1, 63 consuming S2, and 67 consuming SS); 146 patients were considered in the PP population. There were no significant differences in the primary outcomes of the groups consuming S1, S2, or SS. However, the percentage of patients achieving HbA1c < 8% was significantly higher in the S2 group than in the SS group. In the ITT population, the 12-week HbA1c and fructosamine levels were lower in the S1 group than in the SS group and the 4-, 8-, and 12-week fructosamine levels were lower in the S2 group than in the SS group. Although we failed to achieve the primary outcome, it is possible that the Jeju waters improve glycemic control compared with the Seoul tap water in diabetic patients.

## 1. Introduction 

Jeju water is the groundwater of Jeju Island, a volcanic island located in the southernmost part of Korea. It contains a high concentration of vanadium because Jeju Island is made up largely of basalt, an extrusive igneous rock. A vanadium supplement improved the glucose profiles and reduced the insulin requirements in animal models of types 1 and 2 diabetes [1]. In humans, the administration of oral vanadium has improved glucose control in small experimental studies [2, 3].

Worldwide, the prevalence of and healthcare expenditure on diabetes are increasing explosively. Most of the costs associated with diabetes arise from its chronic complications. Therefore, any inexpensive and easy way to improve glycemic control should be developed. Drinking water is a good example of such options.

Therefore, we hypothesized that the daily ingestion of Jeju water would reduce blood glucose levels. We tested this hypothesis in a prospective randomized controlled trial to assess whether Jeju water improved blood glucose control in patients with diabetes better than out-of-island tap water.

## 2. Materials and Methods

### 2.1. Study Design

This trial was a 12-week double-blind, parallel-group, prospective, randomized, controlled trial conducted at a single center on Jeju Island. Approval to conduct the trial was obtained from the Institutional Review Board of Jeju National University Hospital, and all participants provided their written informed consent. The study was performed in accordance with the Declaration of Helsinki.

Eligible patients were randomized to consume one of three types of water. Two test groups drank Jeju water and the control group drank tap water transported from another region. The three types of water were provided by the Jeju Special Self-Governing Province Development Corporation (JPDC, Jeju, Korea). The two kinds of groundwater were collected from Gyorae-ri, Jocheon-eup (S1), and Daepo-dong, Seogwipo-si (S2), on Jeju Island. The control group consumed tap water transported from Gayang-dong, Gangseo-gu (SS), in the city of Seoul. All three waters were filtered through a 0.45 *μ*m pore-sized microfilter (Pall Korea, Seoul, Korea) to remove bacteria. All three waters passed the drinking-water quality testing of Jeju National University Biotechnology Regional Innovation Center and were colorless, odorless, and tasteless. A courier service delivered the Jeju waters or tap water to the subjects from JPDC at intervals of two weeks. Both the researchers and subjects were blinded to the water that each subject drank. The study protocol only allowed the water to be drunk orally, and neither boiling nor cooking was permitted.

After a screening period of 1 week, the eligible patients were required to drink a liter of Jeju water or tap water per day for 12 weeks. The subjects were randomized in proportions of 1 : 1 : 1, using block randomization, into the S1, S2, and SS groups. The patients were then followed up at weeks 4, 8, and 12. The patients were discontinued if they showed poor compliance with the water consumption protocol (<80%), needed to change their doses or regimens of antihyperglycemic medications, were administered agents that influence blood glucose control, suffered serious adverse events, or violated the study protocol. The first patient visit occurred on 25 October 2010 with the final patient visit being on 15 February 2012. No interim analysis was performed until the study visits were completed.

### 2.2. Study Population

We recruited volunteers who had diabetes mellitus and HbA1c levels of ≤9.0%, who had not changed their dose or regimen of antidiabetic drugs in the preceding 12 weeks, and who were between the ages of 20 and 80 years. Patients were excluded if they had any disease that could be exacerbated by drinking water (congestive heart failure, severe hypertension (systolic blood pressure >180 mmHg or diastolic blood pressure >110 mmHg), nephrotic syndrome, renal failure (serum creatinine ≥4.0 mg/dL), liver cirrhosis with ascites, or any edematous disease), an infectious or inflammatory disease, secondary diabetes attributable to drugs, endocrine or pancreatic disease, malignant neoplasm, or an intractable disease. Pregnant women and patients taking steroids or hormones that influence blood glucose levels were also excluded.

### 2.3. Study Assessments

The chemical and physical properties of the waters studied were investigated by JPDC. Each water analysis was performed three times or more. pH and electrical conductivity were measured with the Orion 5-Star (Thermo Scientific, Beverly, MA, USA), based on the electrode method. Water hardness was determined by ethylenediaminetetraacetic acid titration. Anions (F, Cl, NO_3_, and SO_4_) were measured with an ion chromatography system (ICS-2000, Dionex, Sunnyvale, CA, USA). Major minerals (Ca, Mg, Na, K, and Si) were measured with inductively coupled plasma-optical emission spectrometry (ICP-720ES, Varian, Palo Alto, CA, USA). Trace elements (Al, B, Cr, Fe, Mn, V, and Zn) were also measured with inductively coupled plasma-mass spectrometry (ICP-820MS, Varian).

Fasting plasma glucose (FPG), fructosamine, and glycated hemoglobin (HbA1c) were measured at baseline and at 4, 8, and 12 weeks in the double-blind treatment period; fasting plasma C-peptide, creatinine, albumin, aspartate aminotransferase (AST), and alanine aminotransferase (ALT) were determined at baseline and after 12 weeks. Seven-point self-monitored blood glucose (SMBG) profiles (immediately before each meal, 2 h after each meal, and an additional glucose measurement at 22:00) were obtained only on the day before each study visit.

Plasma glucose was determined with the glucose oxidase method on a TBA-200FR chemical analyzer (Toshiba, Tokyo, Japan). Fructosamine was assessed by colorimetry with the Hitachi Modular P (Hitachi, Tokyo, Japan). HbA1c levels were measured with the HLC-723G8 (Tosoh, South San Francisco, CA, USA) by ion-exchange high-performance liquid chromatography. HbA1c standardization was performed by the Korean Association of Quality Assurance for Clinical Laboratory. C-peptide was determined with Modular Analytics E170 electrochemiluminescence immunoassays (Hitachi, Tokyo, Japan). We calculated the homeostasis model assessments of insulin resistance (HOMA-IR) and pancreatic beta-cell function (HOMA-*β*) using plasma C-peptide levels [4], not insulin levels, because substantial numbers of the patients were receiving subcutaneous insulin. The creatinine, albumin, AST, and ALT measurements were made with a TBA-200FR chemical analyzer. 

The primary efficacy parameter was the difference in the proportion of patients achieving good glycemic control (defined as HbA1c < 7.0%) after the consumption of SS, S1, or S2 at week 12 in the per-protocol (PP) population. The secondary efficacy variables included the differences in HbA1c, fructosamine, and FPG after the consumption of SS, S1, or S2 at weeks 4, 8, and 12 in the intention-to-treat (ITT) population. 

Adverse events, physical parameters, and vital signs were monitored at each study visit. All adverse events were assessed by the investigators for their intensity and relationship to the water consumed. Hypoglycemic events were defined according to the report of the American Diabetes Association Workgroup on Hypoglycemia [5].

### 2.4. Statistical Analyses

The sample size was determined based on our unpublished pilot study. The pilot study showed that the proportions of patients with HbA1c < 7.0% were 50.0% and 74.1% for the group consuming Jeju water and the group consuming the Seoul tap water, respectively, in the PP population, with a total withdrawal and dropout rate of 23.5%. Thus, the sample size required for each treatment group was 77.3 subjects (for a two-sided type I error rate of 5% with 80% power); therefore, 232 subjects should be enrolled in total.

The primary efficacy analysis was based on the PP population, which consisted of patients who completed all 12 weeks of treatment and had no reason for exclusion, including a lack of baseline data, a lack of treatment data at weeks 4, 8, or 12, or any protocol violation. Additional efficacy and safety analyses were performed on the ITT population, defined as all the randomized patients who took at least a sip of the study water.

To compare the characteristics of the groups at baseline, the *χ* ^2^ or Fisher's exact test for categorical variables and ANOVA or Kruskall-Wallis test for continuous variables were used. To compare the proportions of patients in the groups who achieved HbA1c < 7% at week 12, a logistic regression analysis was used, with adjustment for baseline HbA1c levels, sex, and the use of a dipeptidyl peptidase-4 (DPP-4) inhibitor. Multiple linear regressions were used to examine the effects of the Jeju waters on HbA1c, fructosamine, and FPG, at weeks 4, 8, and 12, and on BW and WC at week 12. All linear regression models included their baseline levels, sex, and the use of a DPP-4 inhibitor, as described earlier. In both the logistic and linear regression models, SS was used as the reference group and S1 and S2 were represented by dummy variables.

## 3. Results 

### 3.1. Water Properties

The Jeju waters (S1 and S2) were more alkaline and softer and displayed lower electrical conductivity than SS used as the control. S1 and S2 had fewer anions, such as chlorine, nitrate, and sulfate, than SS. In terms of the mineral and trace element contents, S1 and S2 had more silicon dioxide but less calcium, aluminum, iron, and zinc than SS. The vanadium contents of the waters were in descending order: S2 > S1 > SS (Table 1).

### 3.2. Patient Disposition

In total, 233 patients were enrolled in this study, 37 (15.8%) of whom did not participate in the randomized treatment period. These subjects did not participate in the treatment because they withdrew their consent (9.8%) or they were incorrectly enrolled (6.0%). Thus, 196 patients participated in the randomized, double-blind treatment period, and 67, 66, and 63 participants were randomized to consume SS, S1, and S2, respectively. All these patients were included in the ITT population, for the analysis of the secondary efficacy and safety variables.

**Figure 1:**
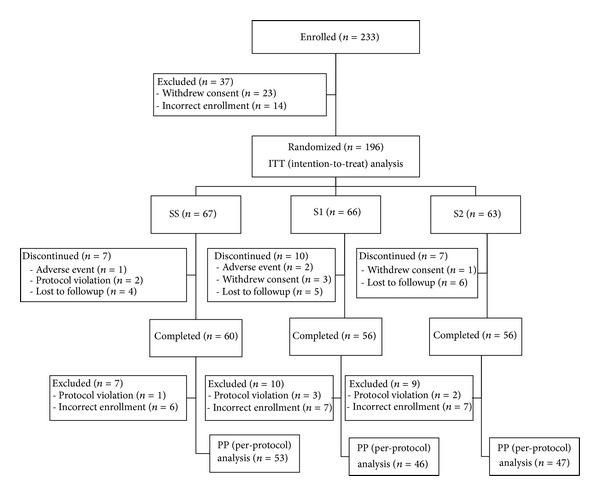
Patient disposition.

After randomization, 172/196 patients completed the 12-week study: 60/67 in the SS group, 56/66 in the S1 group, and 56/63 in the S2 group. The reasons for discontinuation were consent withdrawal and adverse events. Seven patients in the SS group, 10 in the S1 group, and nine in the S2 group were excluded from the PP analysis because they violated the study protocol (change in antidiabetic medication or poor compliance with the protocol) or did not meet the inclusion/exclusion criteria. Consequently, 50 (25.5%) patients in the ITT population were not included in the PP population: 14 (20.9%) in the SS group, 20 (30.3%) in the S1 group, and 16 (25.4%) in the S2 group (not significantly different). The disposition of the patients is shown schematically in Figure 1.

### 3.3. Demographic and Baseline Characteristics

In the ITT population, the demographic and baseline characteristics of the study patients were generally well balanced between the SS, S1, and S2 groups (Table 2). Overall, the patients were predominantly males, with relatively well-controlled diabetes, despite considerable disease durations. Many patients were taking antihypertensive, antihyperlipidemic, and/or antiplatelet agents. Most subjects were being treated with antidiabetic drugs. However, borderline statistically significant differences in sex and the use of DPP-4 inhibitors between the three groups were identified. Therefore, we always adjusted these covariates in the efficacy analyses. There was a significant difference in the baseline characteristics of the PP population in the sex-based distribution across the SS, S1, and S2 groups, whereas the other variables were similar to those of the ITT cohort (data not shown).

### 3.4. Primary Outcome

**Figure 2:**
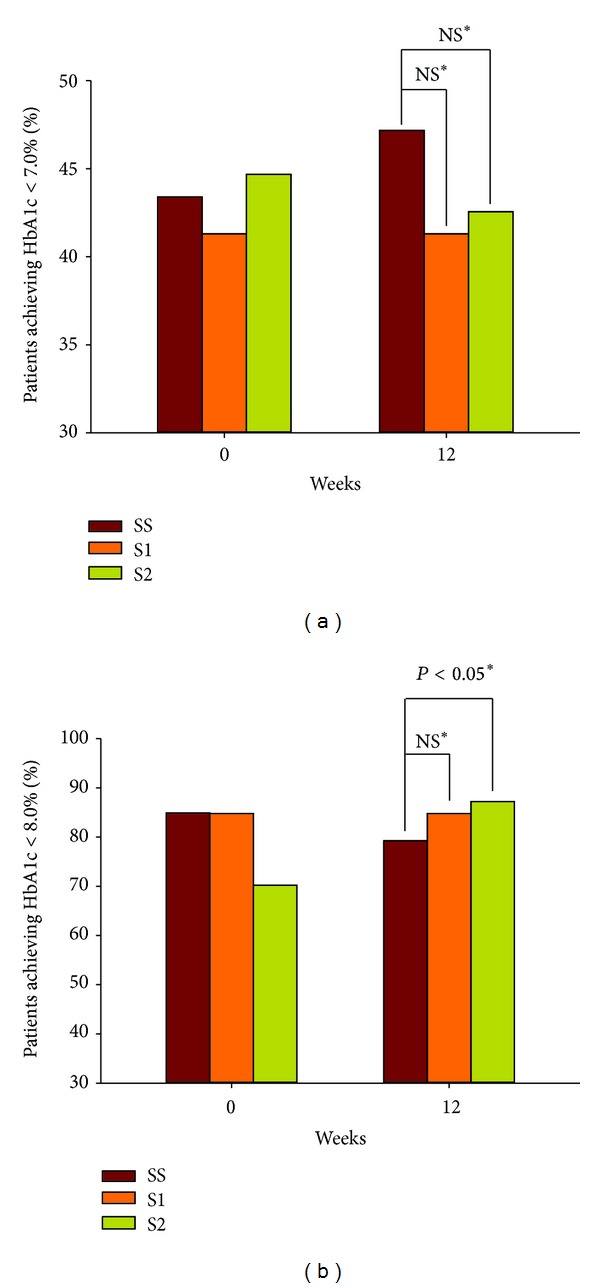
Percentages of patients with HbA1c < 7.0% (a) and <8.0% (b) at the initial and week 12 follow-up visits in the PP population. *Logistic regression analyses (adjusted for baseline differences, sex, and the use of DPP-4 inhibitors). NS: no significant difference.

In the PP population, the proportion of patients with HbA1c < 7.0% at week 12 was 47.2% in the SS group, compared with 41.3% in the S1 group and 42.6% in the S2 group. There was no significant difference in the primary outcomes of the groups consuming Jeju waters and the group consuming the Seoul tap water (Figure 2(a)). However, the percentage of patients achieving HbA1c < 8% at week 12 was significantly higher in the S2 group than in the SS group (87.2% versus 79.2%, resp.) (Figure 2(b)).

### 3.5. ITT Analyses

**Figure 3:**
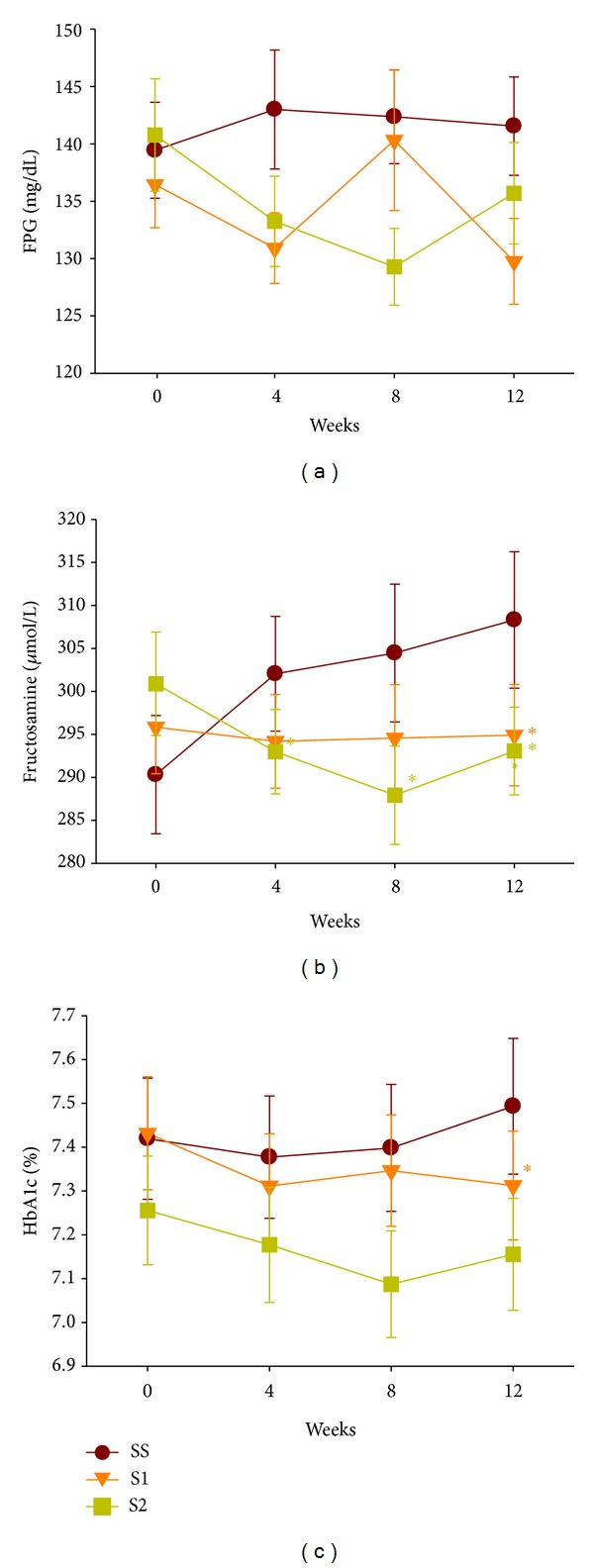
Changes in FPG (a), fructosamine (b), and HbA1c (c) over time in the ITT population. *Linear regression analysis (*P* < 0.05 versus SS. Adjusted for baseline levels, sex, and the use of DPP-4 inhibitors). Each point represents a mean ± SE. FPG, fasting plasma glucose.

**Figure 4:**
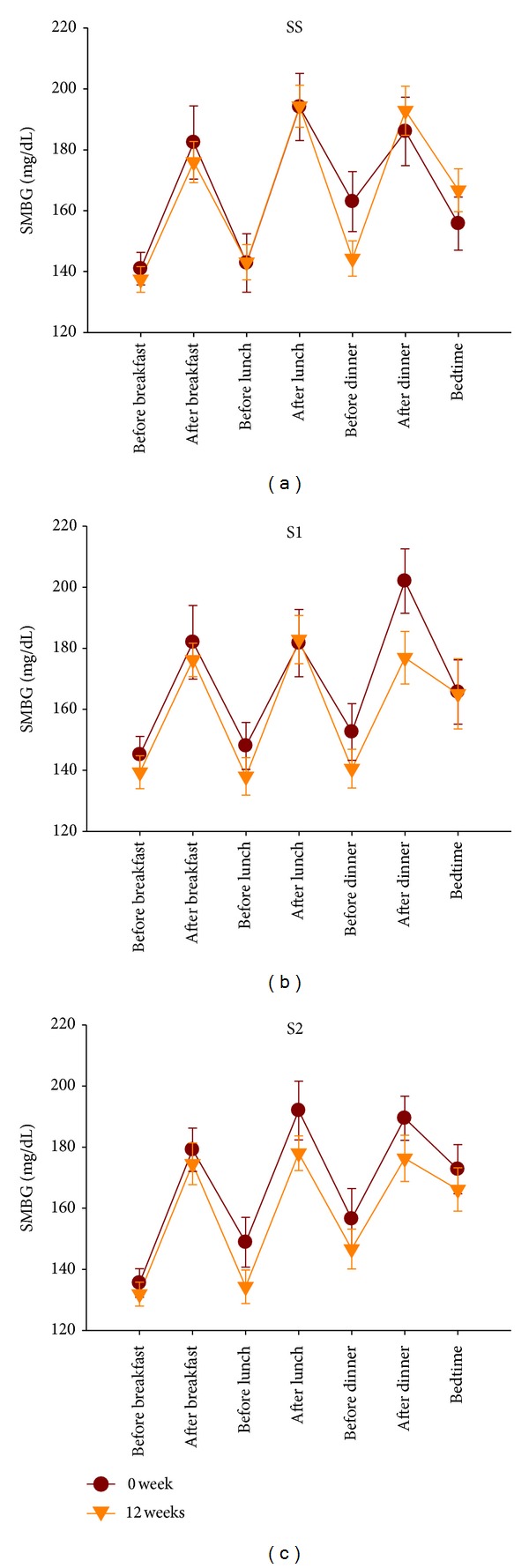
Seven-point self-monitored blood glucose (SMBG) profiles at baseline and week 12 in the SS (a), S1 (b), and S2 (c) groups (ITT population). Each point represents a mean ± SE.

The baseline FPG, fructosamine, and HbA1c levels were similar in the SS, S1, and S2 groups (Table 2). The follow-up FPG levels were not significantly different between the three groups at weeks 4, 8, and 12. However, the fructosamine levels were significantly lower in the S1 group than in the SS group at week 12 and were lower in the S2 group than in the SS group at weeks 4, 8, and 12. The fructosamine levels in the SS group tended to increase during the study, but they showed no change or a slight tendency to decline in the S1 and S2 groups. The HbA1c levels did not differ significantly between the three groups at week 4 or 8, whereas at week 12, the HbA1c levels differed significantly between the S1 and SS groups and differed between the S2 and SS groups with borderline statistical significance (Figure 3 and Table 3). The seven-point SMBG profiles did not differ significantly between baseline and week 12 in all three groups. However, there was a weak decreasing trend in the daytime and evening glucose concentrations in the S2 and S1 groups after the 12-week study period (Figure 4). The plasma C-peptide levels were similar among the three groups at the visit at week 12. HOMA-*β* C-peptide was significantly higher in the S1 group than in the SS group at the end of the study, but HOMA-IR C-peptide did not differ between the three groups (Table 4). Body weight did not differ between the three groups at week 12, but waist circumference differed between the S2 and SS groups with borderline significance (Table 5).

The safety profiles of S1 and S2 were similar to that of SS. The indices of hepatic and renal function did not differ between the three groups at the end of the study (data not shown). Overall, the incidence of adverse events during the 12-week study period did not differ between the three groups. Serious adverse events and study discontinuation because of adverse events were very rare, and the between-group differences were not significant (Table 6).

## 4. Discussion 

Based on the results for the prespecified primary outcome, this trial did not demonstrate that the Jeju waters improved glycemic control in diabetic patients. However, if the HbA1c criterion had been elevated to 8.0%, this study would have met the primary objective. Some of the secondary endpoints, such as the fructosamine and HbA1c levels at certain time points, were slightly reduced in the S1 and S2 groups compared with the SS group. Therefore, although the Jeju waters had no significant effect on the primary outcome of this study, they might have improved glycemic control better than the Seoul tap water. Jeju Island is one of the administrative districts in South Korea. Interestingly, it has the highest prevalence of obesity but the lowest prevalence of diabetes in South Korea [6]. These findings also support our results.

There have been few intervention trials investigating the effects of certain waters on glycemic control in diabetic patients. To our knowledge, the study by Kitta et al. is the only trial to assess the effects of natural water on blood glucose levels in diabetic patients [7]. However, this study was not systematic, reported no detailed data (e.g., the numbers of subjects), and was published in a domestic journal in Japanese. In contrast, our study was a systematic, prospective, parallel-group, randomized, controlled trial with organized clinical data.

There are several potential limitations of this study. The first was the assignment of tap water to the control group. We tried to compare the Jeju waters with the most popularly consumed water in Korea. Therefore, we chose tap water from the city of Seoul, the most populous city in Korea, as the control water. In our study, the Jeju waters showed potential efficacy in improving the glycemic control of diabetic patients compared with the Seoul tap water. However, this effect might not be attributable to the favorable effects of the Jeju waters on glycemic control, but to the harmful effects of the Seoul tap water. As depicted in Table 3, fructosamine tended to increase over time in the control group during the entire study period. However, there was no change or a decreasing trend in fructosamine in the groups that consumed the Jeju waters. However, in this study, we could not determine whether the Jeju waters improved glycemic control or whether the Seoul tap water increased the glucose levels. The obvious finding was that the Jeju waters controlled blood sugar better than the Seoul tap water. To analyze the specific effects of tap, ground, and Jeju waters separately, it will be necessary to perform a clinical trial with three arms: Jeju water, Seoul tap water, and a groundwater from a different region. Second, the differences in blood sugar control stimulated by the two Jeju waters and the Seoul tap water were very small. The size of the differences was probably attributable to the short study period. Most trials that investigate antidiabetic drugs require at least 6 months. However, in our trial, the subjects consumed the study waters for only 12 weeks; consequently, fructosamine, a short-term glycemic index, was reduced more than HbA1c in the Jeju water arms. If our study period had been extended to 6 months or more, there would have been clear differences in the glycemic variables of the S1/S2 and SS groups. Third, the subject population was considerably heterogeneous. Both type 1 and 2 diabetic patients were included, who were receiving various therapies for diabetes. This was because a single center should recruit all subjects for a short period. If our trial targeted a homogeneous group, for example, drug-naïve patients with type 2 diabetes, we would assess more clear effects of Jeju waters on glycemic control.

In this trial, we could not identify the mechanism underlying the effect exerted by Jeju water on glycemic control. It was unclear whether the high vanadium content of the Jeju water lowered the patients' glucose levels. We tried to measure the blood vanadium levels of the study subjects but failed. Jeju water has a lower concentration of vanadium than some volcanic mineral waters, such as Mt Fuji groundwater [7, 8]. We showed that the HOMA-*β* C-peptide, an index of *β*-cell function, was higher in the Jeju water (S1) group than in the Seoul tap water (SS) group at the end of the study. This finding differs from those of previous reports in which vanadium compounds improved glucose control through their insulin-mimetic actions [1]. As shown in Table 1, the Jeju waters were more alkaline than the Seoul tap water. It has been reported that drinking acidic water is associated with an increased risk of type 1 diabetes [9]. The nitrate concentration was lower in the Jeju waters than in the Seoul tap water. Epidemiological data suggest that nitrate intake is related to the development of type 1 and 2 diabetes [10]. Thus, a higher pH and low nitrate could be tentatively proposed as the mechanisms underlying the glycemic improvement achieved by drinking Jeju water. More research is required to determine how Jeju water reduces blood glucose levels compared with Seoul tap water.

## 5. Conclusions

Although this trial did not see a change in the primary efficacy variable, some glycemic variables were more reduced in the groups consuming Jeju waters than in the group consuming Seoul tap water. Therefore, it is possible that Jeju water improves glycemic control in diabetic patients more effectively than Seoul tap water. To confirm that Jeju water exerts a glucose-lowering effect, we must change the primary outcome, include another groundwater in the trial arms, extend the study period, and increase the number of centers involved. The redesigned clinical trial may offer a new alternative treatment for diabetes mellitus.

## Figures and Tables

**Table 1:** Geographic features, physicochemical characteristics, mineral contents, and trace elements of the study waters.

	SS	S1	S2	P*
Type	Tap water	Groundwater	Groundwater	
Region	Gayang-dong, Seoul	Gyorae-ri, Jeju	Daepo-dong, Jeju	
pH	7.4 ± 0.0	7.7 ± 0.0	8.2 ± 0.0	0.025
Electrical conductivity (*μ*s/cm)	130.3 ± 0.2	75.0 ± 1.1	105.5 ± 0.1	0.027
Hardness (mg/L)	39.4 ± 1.5	18.4 ± 0.7	24.3 ± 1.0	0.027
F (mg/L)	0.0 ± 0.0	0.0 ± 0.0	0.1 ± 0.0	0.061
Cl (mg/L)	13.8 ± 1.4	5.9 ± 0.2	5.3 ± 0.0	0.026
NO_3_ (mg/L)	8.9 ± 0.6	1.5 ± 0.1	1.5 ± 0.2	0.044
SO_4_ (mg/L)	9.9 ± 1.4	1.6 ± 0.1	1.7 ± 0.1	0.050
Ca (mg/L)	13.4 ± 1.6	3.2 ± 0.2	2.8 ± 0.1	0.044
K (mg/L)	2.4 ± 0.0	2.2 ± 0.0	4.2 ± 0.1	0.026
Mg (mg/L)	3.1 ± 0.5	2.7 ± 0.1	3.7 ± 0.1	0.249
Na (mg/L)	6.9 ± 0.4	5.7 ± 0.3	10.2 ± 0.5	0.027
SiO_2_ (mg/L)	8.2 ± 0.2	27.0 ± 0.2	26.4 ± 0.4	0.044
V (mg/L)	1.3 ± 0.1	7.7 ± 0.3	20.5 ± 0.6	0.027
Al (*μ*g/L)	26.9 ± 1.8	2.8 ± 0.3	6.3 ± 0.6	0.027
B (*μ*g/L)	9.3 ± 0.6	7.9 ± 1.7	9.7 ± 1.1	0.561
Fe (*μ*g/L)	50.4 ± 8.5	10.6 ± 2.6	12.5 ± 3.6	0.066
Cr (*μ*g/L)	0.9 ± 0.7	0.6 ± 0.4	0.6 ± 0.4	0.860
Zn (*μ*g/L)	14.1 ± 0.7	0.7 ± 0.1	9.3 ± 1.2	0.027
Mn (*μ*g/L)	1.5 ± 0.3	0.1 ± 0.1	0.1 ± 0.1	0.057

Data are expressed as means ± SE from three independent measurements.

*Kruskal-Wallis test.

**Table 2:** Demographic and baseline characteristics of the patients in the ITT population.

Variables	SS (*n* = 67)	S1 (*n* = 66)	S2 (*n* = 63)	P*
Age, years	58.1 ± 1.2	58.9 ± 1.1	59.7 ± 1.0	0.574
Sex, male, %	82.1	63.6	73.0	0.057
Weight, kg	71.5 ± 1.2	68.9 ± 1.4	69.2 ± 1.6	0.339
BMI, kg/m^2^	26.0 ± 0.4	26.0 ± 0.4	25.8 ± 0.5	0.898
Waist circumference, cm	90.5 ± 0.8	89.5 ± 0.9	90.4 ± 1.1	0.702
SBP, mmHg	141.2 ± 1.9	140.3 ± 2.1	142.8 ± 2.3	0.711
DBP, mmHg	82.3 ± 1.4	84.6 ± 1.3	83.9 ± 1.4	0.454
DM duration, years	9.5 ± 0.9	8.1 ± 1.0	8.0 ± 0.8	0.418
FPG, mg/dL	139.4 ± 4.2	136.5 ± 3.8	140.8 ± 4.9	0.768
HbA1c, %	7.4 ± 0.1	7.4 ± 0.1	7.3 ± 0.1	0.575
Fructosamine, *μ*mol/L	290.3 ± 6.9	295.8 ± 5.4	300.9 ± 6.0	0.480
C-peptide, ng/mL	2.0 ± 0.84	2.3 ± 1.04	2.3 ± 1.06	0.248
HOMA-*β* C-peptide	61.9 ± 4.00	74.3 ± 8.70	63.8 ± 3.10	0.268
HOMA-IR C-peptide	1.7 ± 0.1	1.9 ± 0.1	2.0 ± 0.1	0.259
Creatinine, mg/dL	1.1 ± 0.0	1.1 ± 0.0	1.1 ± 0.0	0.973
AST, U/L	25.2 ± 1.3	28.4 ± 2.0	26.3 ± 1.8	0.416
ALT, U/L	30.6 ± 2.2	34.3 ± 3.5	31.1 ± 2.5	0.592
Albumin, g/dL	4.3 ± 0.0	4.3 ± 0.0	4.3 ± 0.0	0.675
Antihypertensive drugs, %	53.7	53.8	51.6	0.961
Lipid-lowering agents, %	52.2	52.3	50.0	0.957
Antiplatelet agents, %	34.3	30.8	32.3	0.908
Antidiabetic regimen				
Sulfonylurea, %	59.7	47.6	57.4	0.346
Biguanide, %	82.1	87.3	77.0	0.328
Thiazolidinedione, %	4.5	7.9	8.2	0.633
*α*-Glucosidase inhibitor, %	1.5	6.3	9.8	0.105
DPP-4 inhibitor, %	9.0	22.2	13.1	0.094
Insulin, %	19.4	9.5	14.8	0.282

Data are expressed as means±SE or frequencies (%).

*ANOVA or *χ* ^2^ test.

BMI: body mass index; SBP: systolic blood pressure; DBP: diastolic blood pressure; FPG: fasting plasma glucose; DM: diabetes mellitus; HOMA: homeostasis assessment model; AST: aspartate aminotransferase; ALT: alanine aminotransferase; DPP-4: dipeptidyl peptidase-4.

**Table 3:** Changes in glycemic variables over time in the ITT population.

Variable	Group	Week 4			Week 8			Week 12		
		*n*	Mean ± SE	P*	*n*	Mean ± SE	P*	*n*	Mean ± SE	P*
FPG (mg/dL)	SS	57	143.0 ± 5.2		60	142.4 ± 4.1		60	141.6 ± 4.3	
	S1	53	130.9 ± 3.1	0.236	56	140.3 ± 6.1	0.872	56	129.8 ± 3.7	0.072
	S2	57	133.2 ± 3.9	0.150	54	129.3 ± 3.3	0.052	56	135.7 ± 4.4	0.466

Fructosamine (*μ*mol/L)	SS	57	302.1 ± 6.7		60	304.5 ± 8.0		60	308.3 ± 7.9	
	S1	53	294.2 ± 5.4	0.471	56	294.6 ± 6.2	0.137	56	294.9 ± 5.9	0.023
	S2	56	293.0 ± 4.9	0.019	53	287.9 ± 5.7	0.015	56	293.1 ± 5.1	0.008

HbA1c (%)	SS	57	7.4 ± 0.1		60	7.4 ± 0.1		60	7.5 ± 0.2	
	S1	53	7.3 ± 0.1	0.610	56	7.4 ± 0.1	0.498	56	7.3 ± 0.1	0.018
	S2	57	7.2 ± 0.1	0.644	54	7.1 ± 0.1	0.153	56	7.2 ± 0.1	0.095

*Linear regression analysis (*P* versus SS, adjusted for baseline levels, sex, and the use of DPP-4 inhibitors).

FPG: fasting plasma glucose.

**Table 4:** Plasma C-peptide, HOMA-*β* C-peptide, and HOMA-IR C-peptide at the week 12 visit in the ITT population.

Variable	Group	Week 12		
		*n*	Mean ± SE	P*
C-peptide (ng/mL)	SS	60	2.1 ± 0.1	
	S1	56	2.2 ± 0.1	0.516
	S2	55	2.2 ± 0.1	0.323

HOMA-*β* C-peptide	SS	60	59.3 ± 3.1	
	S1	55	74.8 ± 5.4	0.010
	S2	55	66.1 ± 3.3	0.296

HOMA-IR C-peptide	SS	60	1.8 ± 0.1	
	S1	55	1.8 ± 0.1	0.345
	S2	55	1.8 ± 0.1	0.333

*Linear regression analysis (*P* versus SS, adjusted for baseline levels, sex, and use of DPP-4 inhibitors).

HOMA: homeostasis model assessment.

**Table 5:** Body weight and waist circumference at the week 12 visit in the ITT population.

Variable	Group	Week 12		
		n	Mean ± SE	P*
Body weight (kg)	SS	60	71.7 ± 1.3	
	S1	55	67.2 ± 1.5	0.239
	S2	56	68.4 ± 1.8	0.896

Waist circumference (cm)	SS	60	90.3 ± 0.9	
	S1	55	88.3 ± 1.1	0.455
	S2	56	88.7 ± 1.2	0.054

*Linear regression analysis (*P* versus SS, adjusted for baseline levels, sex, and the use of DPP-4 inhibitors).

**Table 6:** Adverse events in the ITT population.

Variables	SS		S1		S2		P*
	*n*	%	n	%	n	%	
Any adverse events	15	22.4	13	19.7	8	12.7	0.341
Abdominal pain	1	1.5	0	0.0	0	0.0	1.000
Diarrhea	1	1.5	2	3.0	0	0.0	0.374
Edema	1	1.5	1	1.5	0	0.0	1.000
Hypoglycemia	13	19.4	10	15.2	8	12.7	0.569
Symptomatic	12	17.9	8	12.1	7	11.1	0.522
Asymptomatic	3	4.5	3	4.5	2	3.2	1.000
Severe	0	0.0	1	1.5	0	0.0	0.658

Discontinuation because of adverse events	1	1.5	2	3.0	0	0.0	0.656
Serious adverse events	0	0.0	1	1.5	0	0.0	0.658

*χ ^2^ or Fisher's exact test.
